# The Great Pretender: Unmasking Persistent Inflammation as Whipple's Disease

**DOI:** 10.7759/cureus.115346

**Published:** 2026-08-28

**Authors:** Carolina Martins, Mariana Portugal, Catarina Lucas

**Affiliations:** 1 Internal Medicine, Unidade Local de Saúde (ULS) Coimbra, Coimbra, PRT

**Keywords:** duodenal biopsy, iron-deficiency anemia, systemic inflammation, tropheryma whipplei, whipple disease

## Abstract

Whipple's disease is a rare and chronic multisystem infection caused by *Tropheryma whipplei*. Its protean and often nonspecific clinical manifestations frequently delay diagnosis, increasing the risk of irreversible organ damage. A 54-year-old man was referred for evaluation of persistently elevated inflammatory markers and iron-deficiency anemia. He was initially asymptomatic but subsequently developed intermittent fever, unintentional weight loss, and occasional nausea, vomiting, and diarrhea. A CT revealed retroperitoneal lymphadenopathy. Upper gastrointestinal endoscopy demonstrated diffusely edematous duodenal mucosa with a characteristic pearly appearance. Histopathological examination of duodenal biopsies, supported by PCR testing, confirmed *T. whipplei* infection. Tests for neurological and cardiac involvement showed no abnormalities. The patient was treated with intravenous ceftriaxone followed by oral doxycycline and hydroxychloroquine, resulting in normalization of inflammatory markers, resolution of iron-deficiency anemia, sustained clinical recovery, and complete endoscopic remission. This case illustrates that Whipple's disease may initially present with persistent systemic inflammation and iron-deficiency anemia before other significant symptoms arise. Recognizing these unusual features may help enable earlier diagnosis and treatment, preventing irreversible organ damage.

## Introduction

Whipple's disease is a rare chronic multisystem infection caused by *Tropheryma whipplei*, a Gram-positive actinobacterium. It was first described by George H. Whipple in 1907 in a patient with progressive weight loss, steatorrhea, abdominal symptoms, and arthritis, establishing the first reported case of what he named "intestinal lipodystrophy" [1]. Although it is widespread and asymptomatic carriage is relatively common, only a small number of exposed people develop clinical disease. This is likely due to an underlying defect in the host immune response. Whipple's disease mainly affects middle-aged white men and often presents with migratory arthralgia, diarrhea, weight loss, and abdominal pain [2-5].

Despite these characteristic features, the clinical presentation varies considerably among patients. Constitutional symptoms, isolated laboratory abnormalities, or extraintestinal manifestations may precede gastrointestinal symptoms by months or even years. This can lead to extensive investigation for more common conditions such as malignancy, chronic infections, autoimmune disorders, or inflammatory diseases [3,6,7]. The diagnosis is established by identifying periodic acid-Schiff (PAS)-positive foamy macrophages in duodenal biopsies, with PCR providing confirmatory evidence of the infection [2,5,6].

We present the case of a 54-year-old man who showed persistent systemic inflammation and iron-deficiency anemia before any other symptoms appeared. The diagnosis of Whipple's disease was established after endoscopic evaluation and molecular confirmation, highlighting the importance of considering this rare condition in the differential diagnosis of unexplained inflammatory syndromes.

## Case presentation

A 54-year-old man with a history of cognitive impairment, dyslipidemia, hypothyroidism, and gastritis was referred to the Internal Medicine outpatient clinic for evaluation of iron-deficiency anemia and persistently elevated inflammatory markers for one year. His regular medications included pantoprazole, levothyroxine, and a fixed-dose combination of atorvastatin and ezetimibe. The patient was asymptomatic at presentation, denying abdominal pain or arthralgia and with no constitutional, gastrointestinal, genitourinary, or respiratory complaints. Laboratory findings revealed iron-deficiency anemia (hemoglobin: 11.4 g/dL), markedly elevated inflammatory markers (erythrocyte sedimentation rate: 62 mm/h and C-reactive protein: 9.01 mg/dL), and an elevated serum vitamin B12 level (1455 pg/mL). Autoimmune screening and infectious disease serologies were negative, and the remaining laboratory findings were unremarkable (Table 1).

**Table 1:** Laboratory results since the first evaluation till the end of treatment

Laboratory parameters	First evaluation	Four months of symptoms	Three months of therapy	18 months of therapy	Reference range
Hemoglobin (g/dL)	11.4	8.7	12.5	13.5	12.0-15.6
Mean corpuscular volume (fL)	79.4	77.9	83.6	89.0	80-99
Iron (µg/dL)	44	9	-	78	70-180
Ferritin (ng/mL)	254	110.8	-	43.3	30-300
Transferrin saturation (%)	18	4	-	24	20-40
Folic acid (ng/mL)	4.9	3.3	> 20	-	>3.5
Vitamin B12 (pg/mL)	1455	1054	1170	-	187-883
Erythrocyte sedimentation rate (mm/h)	62	47	33	10	1-20
C-reactive protein (mg/dL)	9.06	8.69	0.16	-	< 0.5
Thyroid-stimulating hormone (mIU/L)	5.0	3.3	4.0	-	0.35-4.94
Free thyroxine (T4) (ng/dL)	0.84	0.89	0.81	-	0.7-1.5
Antinuclear antibodies/extractable nuclear antigens screen	Negative	-	-	-	-
Anti-transglutaminase IgA; IgG (AU/mL)	Negative	-	-	-	-
Gastric parietal cell antibodies (U/mL)	Negative	-	-	-	-
Intrinsic factor antibodies (U/mL)	Negative	-	-	-	-
*Rickettsia conorii *serology	Negative	-	-	-	-
*Coxiella burnetii* serology	Negative	-	-	-	-
Leptospirosis serology	Negative	-	-	-	-
HIV serology	Negative	-	-	-	-
Hepatitis B serology	Negative	-	-	-	-
Hepatitis C serology	Negative	-	-	-	-
Anti-endomysial IgA/IgG	-	Negative	-	-	-
Fecal calprotectin (mg/kg)	-	289	-	-	< 50
Stool culture (*Shigella*, *Salmonella*, *Campylobacter*)	-	Negative	-	-	-
*Clostridium difficile* glutamate dehydrogenase/toxins A and B	-	Negative	-	-	-
Stool parasitology	-	Negative	-	-	-
Total prostate-specific antigen (ng/mL)	-	1.27	-	-	<4

**Figure 1:**
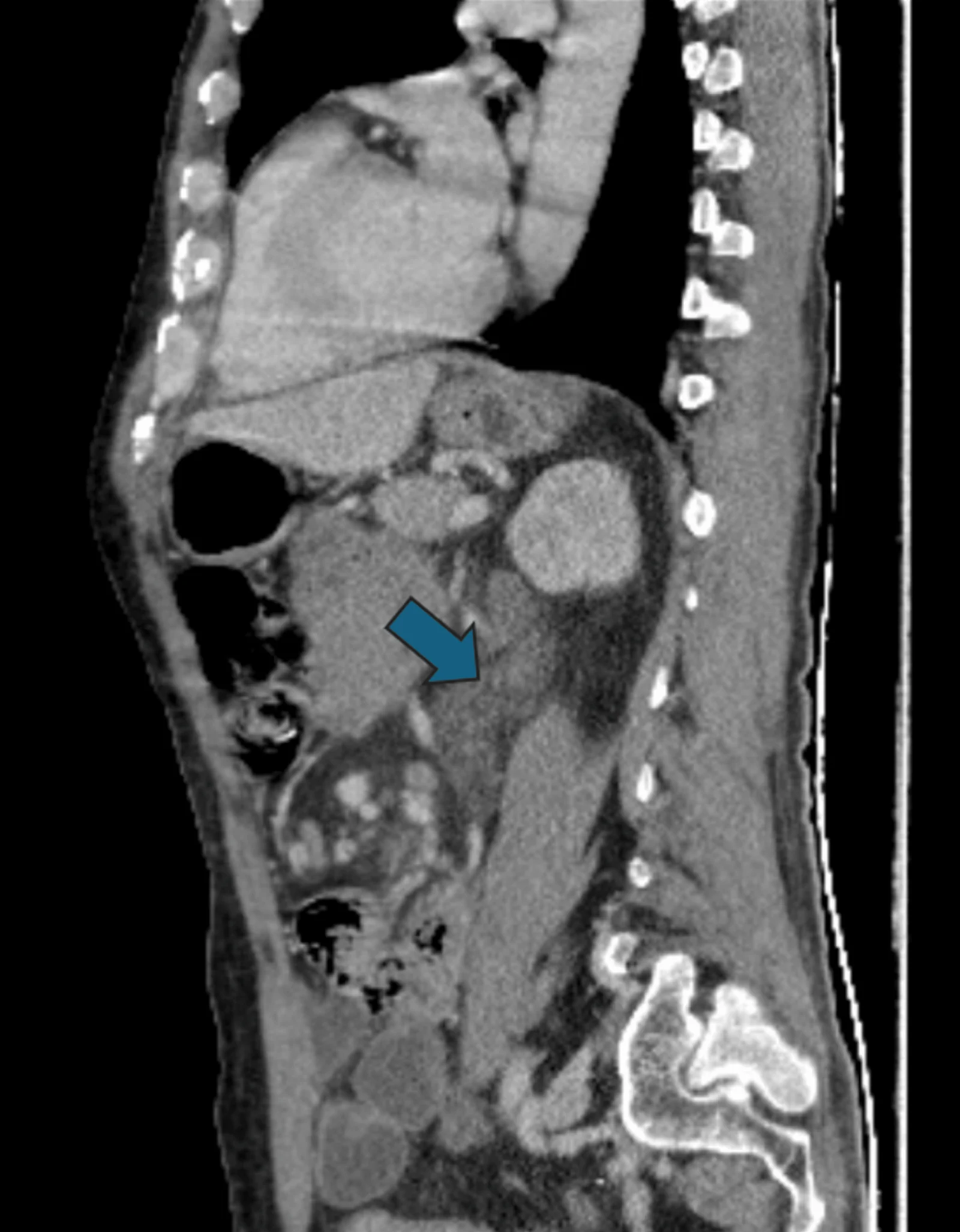
Contrast-enhanced abdominal CT shows bulky retroperitoneal lymphadenopathy (blue arrow)

Over the following four months, the patient's clinical condition evolved. He developed intermittent fever, unintentional weight loss (not quantified), and occasional episodes of nausea, vomiting, and diarrhea. Repeat laboratory testing demonstrated worsening anemia (hemoglobin 8.7 g/dL), marked iron deficiency (iron 9 µg/dL), as well as persistently elevated inflammatory parameters. Further investigation was conducted, including fecal calprotectin, stool cultures, and parasitological examinations, as well as serological testing for celiac disease, all of which were normal (Table 1). Given the sustained systemic inflammation and the emergence of constitutional and gastrointestinal symptoms, a whole-body CT scan, an endoscopy, and colonoscopy were requested. The CT scan revealed retroperitoneal lymphadenopathy with homogeneous low attenuation suggestive of adipose infiltration, without additional significant abnormalities (Figure 1).

**Figure 2:**
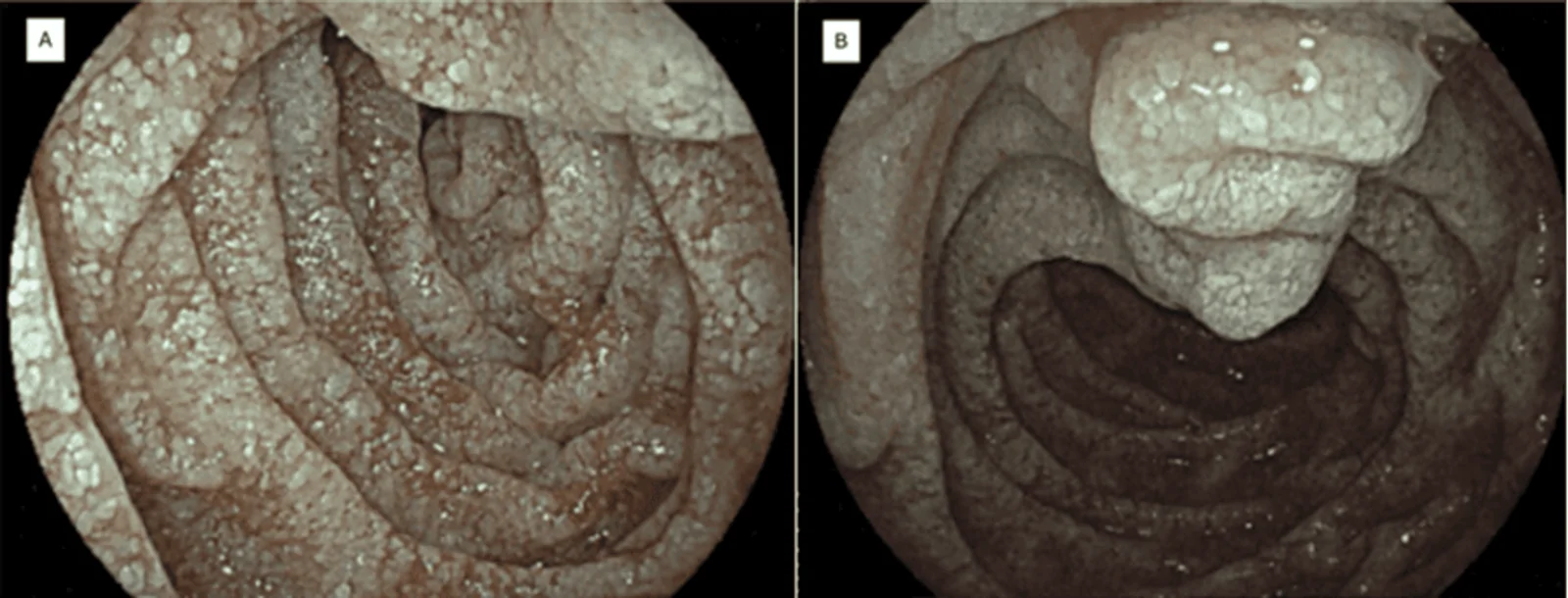
Endoscopic findings Endoscopic appearance of the duodenum demonstrates diffuse pearly, edematous mucosa (A) with prominent villi (B).

The upper gastrointestinal endoscopy then demonstrated diffusely pearly, swollen duodenal mucosa (Figure 2), raising suspicion of Whipple's disease. A histopathological examination of several duodenal biopsies revealed PAS-positive, CD68-positive foamy macrophages in the tissue, while Ziehl-Neelsen staining was negative. The PCR confirmed the diagnosis of infection by *T. whipplei*. The colonoscopy showed no abnormalities. Following diagnostic confirmation, further tests were performed to assess for extraintestinal involvement. Brain CT and cerebrospinal fluid analysis showed no signs of central nervous system disease, and transthoracic echocardiography ruled out cardiac involvement.

Treatment was initiated with an induction course of intravenous ceftriaxone (2 g once daily) for two weeks, followed by oral doxycycline (200 mg once daily) in combination with hydroxychloroquine (600 mg once daily), with a planned total treatment duration of 18 months. Three months after treatment initiation, inflammatory markers had normalized, and the iron-deficiency anemia had resolved. Follow-up upper gastrointestinal endoscopies performed at six months and at completion of therapy demonstrated complete endoscopic remission, accompanied by sustained clinical recovery and normalization of biochemical parameters (Table 1).

## Discussion

Whipple's disease remains a rare diagnosis due to its wide and often nonspecific clinical spectrum. Although chronic diarrhea, weight loss, abdominal pain, and migratory arthralgia constitute the classic clinical presentation, these symptoms can be incomplete or appear late in the disease course, delaying diagnosis and leading to investigations for more common conditions [2,3,6,7]. Our patient exemplifies this diagnostic challenge. His initial presentation included persistent systemic inflammation and iron-deficiency anemia without significant symptoms. This led to an extensive evaluation for infectious and autoimmune causes. The subsequent development of constitutional symptoms led to abdominal imaging, which revealed retroperitoneal lymphadenopathy and prompted upper gastrointestinal endoscopy. Retroperitoneal lymphadenopathy can occur in Whipple's disease, but it is also a nonspecific finding that should be evaluated in the proper clinical context alongside endoscopic and histopathological results [2]. While the characteristic pearly, swollen appearance of the duodenal mucosa is not pathognomonic, it served as an important clue prompting tissue biopsy.

The diagnosis was made by identifying PAS-positive foamy macrophages in duodenal biopsies and confirmed by PCR for T. whipplei. Histopathology is essential for diagnosis, while PCR enhances accuracy, especially in cases with atypical clinical presentations or unclear histological findings [2,5,6]. After diagnosing Whipple's disease, evaluation for neurological and cardiac involvement is recommended, as these manifestations may influence prognosis and therapeutic management, even in asymptomatic patients [2,4]. In our patient, these assessments were normal, allowing for prompt treatment before any irreversible organ damage occurred.

Regarding treatment, we followed a commonly used protocol that consists of an initial two-week course of intravenous ceftriaxone (2 g once daily) followed by prolonged oral therapy for at least 12 months. Trimethoprim-sulfamethoxazole remains the most commonly used oral antimicrobial regimen, typically administered at a dose of 160/800 mg twice daily [2,3,4,8]. In our patient, however, an alternative regimen consisting of doxycycline (200 mg/day) combined with hydroxychloroquine (600 mg/day) was selected due to a documented allergy to trimethoprim-sulfamethoxazole. Recent evidence suggests that an exclusively oral regimen may achieve outcomes comparable to those of treatment protocols incorporating an initial intravenous induction phase [8].

The quick normalization of inflammatory markers, resolution of anemia, sustained clinical improvement, and complete endoscopic remission seen during follow-up highlight the favorable response that can be achieved with appropriate antimicrobial therapy. This case demonstrates that persistent systemic inflammation and iron-deficiency anemia may precede the development of prominent gastrointestinal symptoms. Identifying this unusual presentation and pursuing endoscopic evaluation when initial tests are inconclusive can lead to earlier diagnosis and better outcomes in this rare but treatable disease.

## Conclusions

Whipple’s disease should remain a possible diagnosis when more common conditions cannot adequately explain persistent inflammatory abnormalities and iron-deficiency anemia. This case highlights that an atypical initial presentation should not preclude the diagnosis and that diagnostic suspicion should be reconsidered as new clinical findings emerge. Ultimately, this case reinforces the importance of maintaining a broad diagnostic perspective in patients with unexplained and persistent abnormalities. Recognizing less typical presentations of rare diseases such as Whipple’s disease can facilitate appropriate investigation and, consequently, earlier diagnosis, prompt appropriate antimicrobial therapy, and prevent irreversible organ damage.
